# Effectiveness of enhanced supervision, health education and environmental improvement interventions for injuries among children aged 6–17 in Shijiazhuang

**DOI:** 10.3389/fpubh.2026.1733074

**Published:** 2026-02-20

**Authors:** Qiwei Chen, Cong Gao, Huichao Zhang, Yufan Duan, Xiao Deng, Danping Geng, Xinyan Ma

**Affiliations:** 1 School of Public Health, Hebei Medical University, Shijiazhuang, China; 2 Shijiazhang Center for Disease Control and Prevention, Shijiazhang, China; 3 National Center for Chronic and Noncommunicable Disease Control and Prevention, Chinese Center for Disease Control and Prevention, Beijing, China

**Keywords:** child supervision, children, environmental improvement, health education, randomized controlled trial, unintentional injury

## Abstract

**Background:**

Injuries represent a significant public health concern, ranking as the primary cause of mortality among children in China. The 5E strategy is one of the most frequently employed strategies for injury prevention, yet the efficacy of a single prevention strategy is often eclipsed by that of a comprehensive intervention strategy. The present study employed a comprehensive intervention approach, encompassing enhanced supervision, health education and environmental improvement, to evaluate the efficacy of such interventions in preventing injuries among children aged 6–17.

**Methods:**

The study population consisted of children aged 6–17 years. A stratified cluster sampling method was adopted to select 6 primary schools, 6 junior schools, and 4 senior high schools from Yuanshi County and Lingshou County respectively, which were then randomly assigned to the intervention group and the control group. The intervention group received a one-year comprehensive injury prevention intervention (January to December 2019), included team-based efforts to strengthen child supervision, with daily visits during school days and weekly visits during holidays; monthly health education sessions on injury prevention; and biannual home-school-community environmental inspections and improvements. The control group received no intervention.

**Results:**

The intervention group showed a 0.13% decrease in injury incidence following the intervention, while the control group exhibited a 2.00% increase in injury incidence during follow-up compared to baseline. Analysis using the difference-in-differences method indicated that the intervention reduced injury incidence by 2.13% (95% CI: [0.506, 0.809], *p* < 0.001). Specifically, children’s injury-related knowledge scores increased by 2.830 points (95% CI: [2.659, 3.001], *p* < 0.001), and their injury-related protective behavior scores improved by 4.573 points (95% CI: [4.308, 4.839], *p* < 0.001). For parents, injury-related knowledge scores rose by 3.076 points (95% CI:[2.804, 3.347], *p* < 0.001), and their injury-related protective behavior scores increased by 1.358 points (95% CI: [0.543, 2.174], *p* = 0.001). No significant changes were observed in injury-related belief scores (children: *p* = 0.354; parents: *p* = 0.576) or injury-related risky behavior scores (children: *p* = 0.961; parents: *p* = 0.426) among either children or parents.

**Conclusion:**

Interventions that integrate childcare, health education and environmental improvements shown a positive impact on the reduction of childhood injuries, the enhancement of injury prevention knowledge, and the promotion of injury prevention behaviors.

## 1 Background

Unintentional injury has emerged as a critical public health challenge worldwide, primarily attributed to its status as the leading contributor to mortality among children and adolescents (1, 2). Beyond the profound health impacts, it also inflicts substantial disease burden and economic strain on governments, communities, and individual families (3). In China, injury ranks first as the cause of death for children and adolescents aged 1–19 years (4). According to official statistics, the child injury mortality rate in 2021 was 10.28 per 100,000, representing a 7.1% reduction compared with the prior year (5). While time-series studies have documented a gradual downward trend in the incidence and mortality of childhood unintentional injuries (6, 7), the absolute burden remains significant, highlighting the urgent need for implementing targeted and effective preventive measures to further mitigate the occurrence of unintentional injuries in this population.

Mounting evidence indicates that unintentional injuries can be effectively prevented and managed through targeted interventions (8). Globally, the 5E strategy has emerged as a standard framework for injury prevention, comprising five key components: Education, Environmental modification, Enforcement of safety regulations, Engineering improvements, and Evaluation of intervention outcomes. Among these, educational interventions are the most frequently implemented. For instance, Moore et al. (9) enhanced parental knowledge of burn first aid through educational video-based interventions, and Feng et al. (10) successfully reduced injury risks in children aged 0–3 years by providing parent-focused educational programs via social networking platforms. Beyond education, environmental modifications have been shown to improve safety: household environmental upgrades enhance home safety (11–13), and road infrastructure improvements significantly decrease traffic injury rates (14). Additionally, legislation and stricter enforcement are widely acknowledged as critical and effective tools for injury prevention and control. Niu et al. (15) found that 98.5% of participants perceived legislation as a key driver for increasing child safety seat utilization and reducing traffic injuries; similarly, Shafiq et al. (16) reported that 34.2% of Malaysian parents endorsed legislative mandates for Child Restraint System (CRS) use. Despite the proven efficacy of individual 5E components, the multifactorial etiology of injuries means that single-strategy interventions typically produce modest or inconsistent effects, highlighting the need for comprehensive, multi-component approaches.

As a foundational theoretical framework for health behavior interventions, the Knowledge-Attitude-Behavior (KAP) model has been widely adopted as a critical tool in global child injury prevention research. By constructing a logical chain that connects knowledge acquisition, attitude transformation, and behavioral adoption, the model offers robust scientific rationale for designing interventions to reduce childhood injury rates. Drawing on this theoretical basis, the present study integrated the KAP model into child injury prevention interventions. First, we assessed the baseline status of injury prevention KAP among 6-17-year-old children. Subsequently, we implemented targeted, multi-component comprehensive interventions, including strengthened child supervision, tailored health education programs, and environmental improvement initiatives. We further evaluated the intervention effectiveness across different age subgroups. The primary objectives of this study are to enhance children’s injury prevention knowledge, positive attitudes, and protective behaviors; reduce the overall childhood injury incidence rate; and provide empirical evidence to inform the formulation of targeted child injury prevention policies and practice guidelines.

## 2 Methods

### 2.1 Study design

This study was carried out in Yuanshi and Lingshou counties of Shijiazhuang City, Hebei Province, China. The two counties are comparable in geographical size, economic development level, infrastructure conditions, and other essential indicators.

Phase one involved conducting baseline surveys on injury incidence and injury prevention KAP among children aged 6–17 years in both counties from January to August 2018. Phase two implemented a one-year (January to December 2019) comprehensive child injury intervention program. Phase three involved conducting a follow-up survey (March 2020 to December 2022) to reassess injury incidence rates and KAP regarding injury prevention among the same target population, thereby evaluating the effectiveness of the comprehensive injury prevention intervention.

### 2.2 Sample size

Using PASS 11.0 software, select the formula “Tests for two proportions in a cluster-randomized design” to estimate the sample size. Specific parameter settings are as follows: significance level *α* = 0.05, statistical power 1 − *β* = 0.8; set the number of participants in each intervention and control group to 600, with an equal number of groups in both intervention and control groups. Based on prior research, the estimated injury incidence rate in the control group is 16.5% (17), and the estimated injury incidence rate in the intervention group after implementation is 8.0%; the intraclass correlation coefficient (ICC) is 0.03 (18). The final calculation indicates that 16 groups of 600 participants each are required for both the intervention and control groups, totaling 9,600 participants. Accounting for a 10% dropout rate, the required sample size is 10,667 participants.

### 2.3 Participants

The selection of children followed specific inclusion and exclusion criteria. The inclusion criteria were: (1) aged between 6 and 17 years; (2) permanent residents of intervention or control counties; (3) able to read; (4) willing to sign written informed consent and participate in the study. The exclusion criteria were: (1) children with disabilities; (2) unable to read; (3) unwilling to sign informed consent.

### 2.4 Randomization

Participants were stratified by educational stage into primary, junior, and senior high school students. Schools were designated as the cluster sampling units for each educational stratum. Prior to sampling, all eligible schools in Yuanshi County and Lingshou County were coded with unique identifiers. A random number table method was employed to randomly select 6 primary schools, 6 junior high schools, and 4 senior high schools from each county. The selected schools were then randomly assigned to either the intervention group or the control group to ensure baseline comparability between groups. Baseline enrollment included 11,797 children. Follow-up analysis included 10,616 participants, with a loss to follow-up rate of 10%.

### 2.5 Interventions

#### 2.5.1 Parent-led supervision of children’s participation

The school established a dual-tier supervision system to prevent childhood injuries both on campus and during holidays. Within school premises, injury supervision teams were formed at the class group level, with pupils taking turns to patrol during breaks each day to monitor hazardous behaviors that could lead to falls. During the summer holidays, in collaboration with village committees, injury supervision volunteer teams were formed based on pupils’ residential areas. Parents served as team leaders, guiding pupils to monitor dangerous behaviors such as falls and road traffic injuries on a weekly basis, with feedback on supervision outcomes provided to teaching staff. The intervention was implemented from January to December 2019.

#### 2.5.2 School-CDC collaborative health education initiatives

The school conducted monthly safety and health education sessions focusing on preventing common childhood injuries such as falls, burns and scalds, and road traffic injuries. Each session lasted no <1 h and was delivered by trained teaching staff. Throughout each term, child safety knowledge was disseminated to parents via parent-teacher meetings and parent WeChat groups. Utilizing the official WeChat public accounts of Shijiazhuang CDC and Yuanshi County CDC, monthly articles promoting child injury prevention knowledge were published. Interactive prize-winning quizzes on injury prevention knowledge were conducted biannually. The intervention was implemented from January to December 2019.

#### 2.5.3 Child-centered hazardous environment remediation

Health advisers comprising CDC and township staff organized parents to conduct home hazard identification and modification activities using household screening checklists. These identify risks such as falls and scalding injuries, providing guidance for modifications with annual intervention frequency. Schools employed trained teachers to conduct campus hazard assessments using screening checklists at a frequency of once per term. Findings were relayed to relevant personnel for modifications and posting of warning notices. Community hazard identification relied on holiday injury surveillance volunteer teams conducting child-perspective hazard environment photography activities to identify injury-related risks. Supplementary Tables S1, S2 outlines the checklist items.

### 2.6 Outcome measures

#### 2.6.1 Primary outcome

The primary outcome was the incidence rate of unintentional injuries among children and adolescents in the 12 months prior to the distribution of the questionnaire. An injury event is defined as an occurrence that meets any of the following criteria: The child received medical treatment and was diagnosed with a specific type of injury; The child was absent from work, school, or rest for more than 1 day due to injury (19).

#### 2.6.2 Secondary outcome

Secondary outcome measures include injury prevention KAP scores, with separate questionnaires designed for parents and children. Given cognitive limitations among children in grades 1–3 of primary school, who may struggle to accurately comprehend questionnaire content and possess insufficient memory and recall abilities, parent-completed versions ensure higher reliability. Children in grade 4 of primary school and above, as research subjects, must complete the questionnaires independently. Supplementary Tables S3, S4 list questionnaire items. For injury prevention knowledge and belief items, correct responses receive one point, while incorrect responses receive zero points. Injury-related behaviors are categorized into protective behaviors and risky behaviors, both scored based on frequency of occurrence (never—1, rarely—2, sometimes—3, often—4, always—5, not applicable—0). Higher scores are desirable for protective behaviors, while lower scores are desirable for risk behaviors.

### 2.7 Statistical analysis

Statistical analysis was performed using SPSS 25.0 software. Categorical variables were analyzed using the chi-square tests, while continuous variables (means ± standard deviation) were compared between groups using the *t*-tests. Injury occurrence was treated as a binary outcome variable (assigned values: 0 = no injury, 1 = injury). The frequency and percentage of injury incidence among children in both groups at baseline and during follow-up were described. A mixed-effects logistic regression model was employed to analyze changes in injury incidence rates between the intervention group and the control group before and after the intervention. The model was constructed as follows:


$$\begin{array}{l} \text{logit}(P(\text{Injury}=1))=\beta_{0}+\beta_{1}{\text{Treat}}_{i}+\beta_{2}{\text{Post}}_{t}\\ +\beta_{3}({\text{Treat}}_{i}\times {\text{Post}}_{t})+\beta_{4}X_{\mathrm{it}}+\varepsilon_{\mathrm{it}} \end{array}$$

Changes in KAP scores between the intervention and control groups before and after the intervention were analyzed using a difference-in-differences (DID) model. The model is constructed as follows:


$$Y_{i}=\beta_{0}+\beta_{1}\text{Treat}+\beta_{2}\text{Post}+\beta_{3}(\text{Trea}t_{i}\times \mathrm{Pos}t_{t})+\beta_{4}X_{i}+\varepsilon_{\mathrm{it}}$$

Wherein, *Y_it_* represents the KAP score of the *i*-th subject at time *t*; *Treat_i_* is the group variable (intervention group = 1, control group = 0); *Post_t_* is a time variable (post-intervention = 1, pre-intervention = 0); *Treat_i_ × Post_t_* represents the interaction term, with *β_4_* as its coefficient, indicating the net effect of the intervention; *X_it_* denotes the covariate (child’s gender) to control confounding factors. The t-test was used to judge the significance of coefficient.

## 3 Results

### 3.1 Socio-demographic characteristics

**Figure 1:**
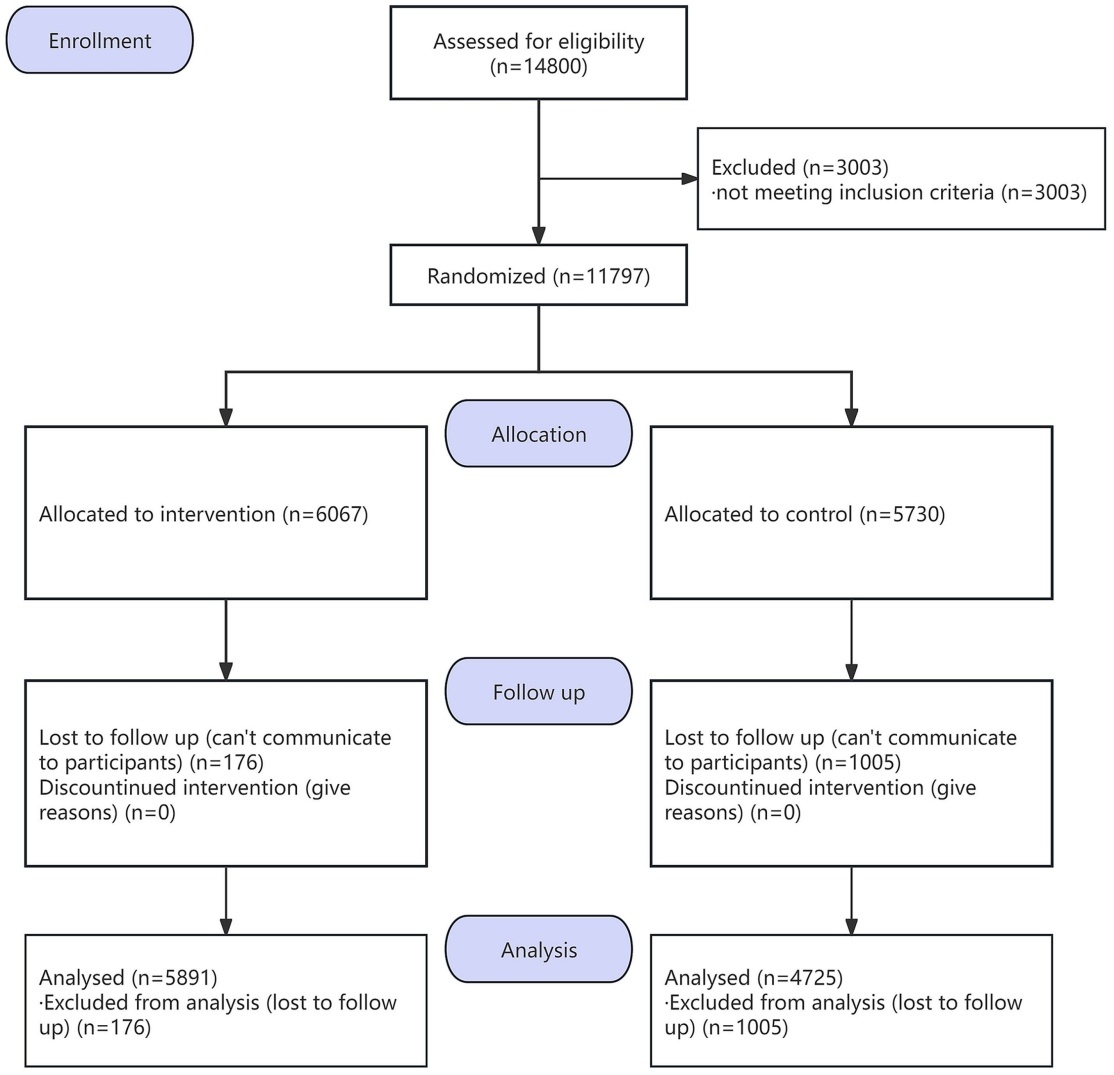
Study flow diagram.

At baseline, we collected 11,797 valid questionnaires which included 6,067 in intervention group and 5,730 in control group. For the follow-up survey, we collected 10,616 valid questionnaires, including 5,891 in intervention group and 4,725 in control group. Figure 1 presents the flow diagram.

This study compared the socio-demographic characteristics between intervention group and control group. There was no statistical difference in gender between the two group of children (*p* = 0.274, *p* = 0.078). There were significant differences in children’s grade and age at baseline and follow-up (all *p <* 0.001) (Table 1).

**Table 1:** Socio-demographic characteristics of sample.

Variable	Baseline		Follow-up	
	Intervention group	Control group	Intervention group	Control group
Children’s gender, *n* (%)				
Male	3,120 (51.43)	2,889 (50.42)	3,035 (51.51)	2,353 (49.80)
Female	2,947 (48.57)	2,841 (49.58)	2,856 (48.47)	2,372 (50.20)
Chi-square values	1.195		3.105	
*p*-value	0.274		0.078	
Children’s grade, *n* (%)				
Grades 1–3	1873 (30.87)	1,268 (22.13)	1,697 (28.81)	1,076 (22.77)
Grades 4–6	1,506 (24.82)	1,032 (18.01)	1,506 (25.56)	1,032 (21.84)
Junior grades	1,163 (19.17)	2,042 (35.64)	1,163 (19.74)	1,229 (26.01)
Senior grades	1,525 (25.14)	1,388 (24.22)	1,525 (25.89)	1,388 (29.38)
Chi-square values	443.308		109.109	
*p*-value	<0.001		<0.001	
Children’s age, mean ± SD	11.31 ± 3.66	11.99 ± 3.26	11.90 ± 3.27	12.63 ± 3.36
*Z*-value	10.782		−12.495	
*p*-value	<0.001		<0.001	

SD, standard deviation.

### 3.2 Unintentional injury incidence

As shown in Table 2, the unintentional injury incidence in the intervention group decreased non-significantly from the baseline of 5.82% to the follow-up of 5.69% (*χ^2^* = 0.096, *p* = 0.757), but the injury incidence in the control group increased significantly from 4.07 to 6.07% (*χ^2^* = 22.087, *p* < 0.001). The change in injury incidence rate was −0.13% in the intervention group and 2.00% in the control group. Calculated using the DID, the net intervention effect was −2.13% (95% CI: [0.506, 0.809], *p* < 0.001).

**Table 2:** The effectiveness of primary outcome.

Group	Stage	Injury incidence (*n*, %)	Within-group intervention effect (*χ^2^*/*p*)	*β^a^*	95%CI	*p* value
Intervention group	Baseline	353 (5.82)	0.096/0.757	−0.447	[0.506, 0.809]	<0.001
	Follow-up	335 (5.69)				
Control group	Baseline	233 (4.07)	22.087/<0.001			
	Follow-up	287 (6.07)				

^a^β is the coefficient of interaction term.

### 3.3 Children’s knowledge, beliefs, and behaviors

**Figure 2:**
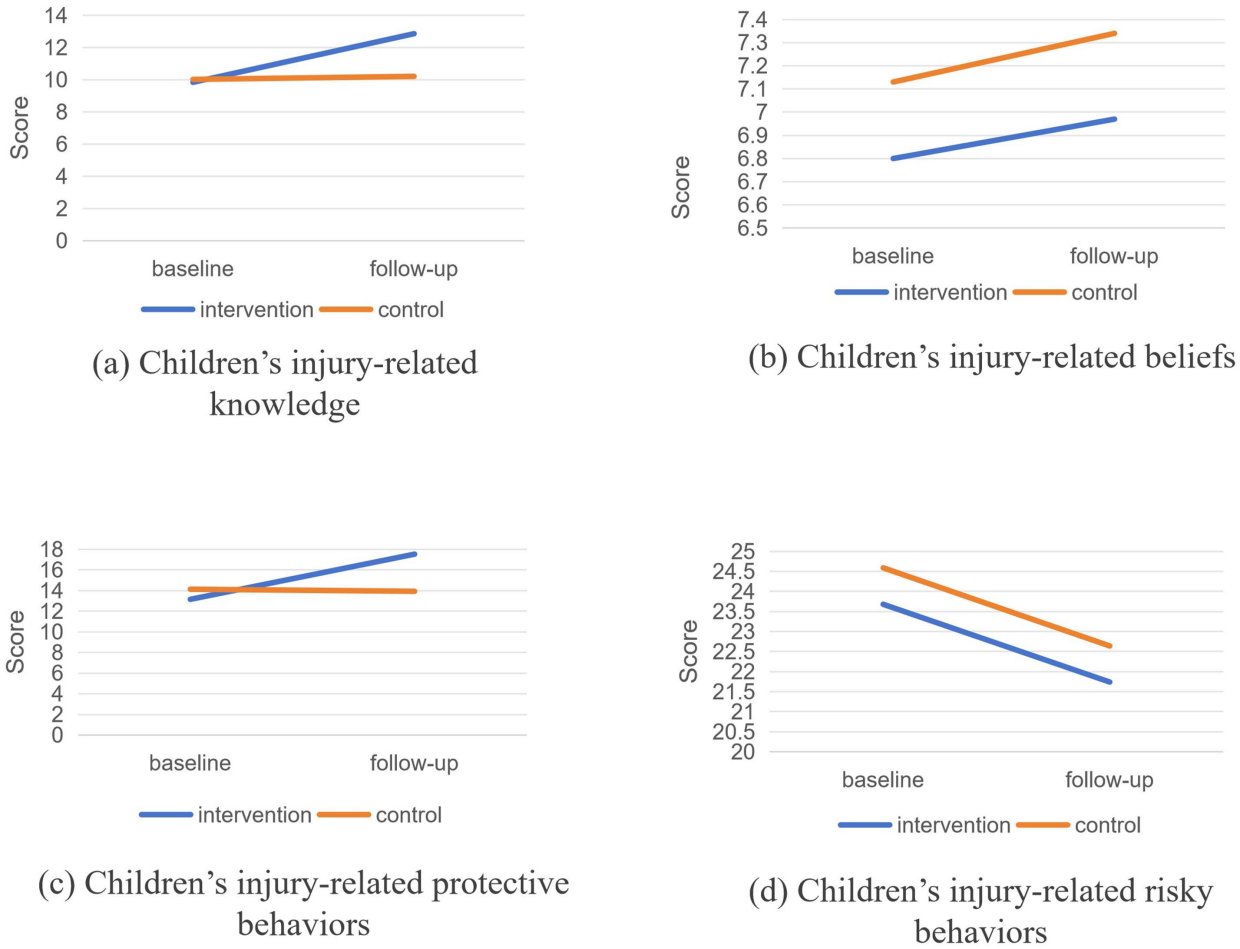
Comparison of baseline and follow-up between children in the intervention and control groups.

As Table 3 and Supplementary Table S5 demonstrated, statistically significant changes were observed in the intervention group children’s scores for injury-related knowledge, beliefs, protective behaviors, and risky behaviors both before and after the intervention (all *p* < 0.001). Similarly, statistically significant changes were also noted in the control group children’s scores for knowledge (*p* < 0.001), beliefs (*p* < 0.001), protective behaviors (*p* = 0.026), and risky behaviors (*p* < 0.001). Comparing the intervention and control groups, DID analysis revealed a 2.830-point improvement in children’s injury-related knowledge (95% CI: [2.659, 3.001], *p* < 0.001) and 4.573-point improvement in protective behavior (95% CI: [4.308, 4.839], *p* < 0.001). Changes in belief scores (*β* = −0.042, 95% CI: [−0.132, 0.047], *p* = 0.354) and risky behavior scores (*β* = 0.008, 95% CI:[−0.328, 0.345], *p* = 0.961) were not statistically significant. (Figure 2).

**Table 3:** The effectiveness of children’s secondary outcome.

Variable	Intervention group		Control group		*β^a^*	95%CI	*p* value
	Baseline	Follow-up	Baseline	Follow-up			
Injury-related knowledge, mean ± SD							
Children	9.84 ± 2.86	12.86 ± 3.86	10.02 ± 2.44	10.21 ± 2.38	2.830	[2.659, 3.001]	<0.001
Primary school	9.44 ± 2.79	12.96 ± 3.65	10.57 ± 2.91	9.94 ± 2.54	4.147	[3.821, 4.473]	<0.001
Junior school	9.12 ± 2.83	10.87 ± 3.09	9.77 ± 2.38	9.69 ± 2.30	1.837	[1.565, 2.109]	<0.001
Senior high school	9.02 ± 2.67	14.88 ± 3.68	9.99 ± 2.06	10.64 ± 2.24	5.212	[4.950, 5.473]	<0.001
Injury-related beliefs, mean ± SD							
Children	6.80 ± 1.82	6.97 ± 1.44	7.13 ± 1.54	7.34 ± 1.50	−0.042	[−0.132, 0.047]	0.354
Primary school	6.80 ± 1.71	7.12 ± 1.34	7.06 ± 1.47	7.02 ± 1.49	0.351	[0.189, 0.513]	<0.001
Junior school	6.67 ± 1.72	6.80 ± 1.58	7.04 ± 1.60	7.19 ± 1.48	−0.023	[−0.184, 0.138]	0.783
Senior high school	6.88 ± 1.98	7.02 ± 1.35	7.30 ± 1.50	7.60 ± 1.47	−0.169	[−0.318, −0.020]	0.026
Injury-related protective behaviors, mean ± SD							
Children	13.15 ± 3.75	17.53 ± 5.78	14.13 ± 4.08	13.93 ± 4.40	4.573	[4.308, 4.839]	<0.001
Primary school	13.17 ± 4.11	19.13 ± 5.42	15.84 ± 4.40	14.67 ± 4.65	7.139	[6.633, 7.645]	<0.001
Junior school	13.68 ± 3.44	16.02 ± 5.29	13.74 ± 3.95	13.18 ± 4.03	2.906	[2.466, 3.346]	<0.001
Senior high school	12.75 ± 3.54	17.76 ± 6.16	13.43 ± 3.67	13.91 ± 4.37	4.540	[4.099, 4.980]	<0.001
Injury-related risky behaviors, mean ± SD							
Children	23.68 ± 5.95	21.74 ± 5.61	24.59 ± 6.45	22.64 ± 5.65	0.008	[−0.328, 0.345]	0.961
Primary school	22.24 ± 5.60	21.50 ± 5.67	20.72 ± 4.99	20.79 ± 4.88	−0.801	[−1.374, −0.228]	0.006
Junior school	24.96 ± 6.00	21.79 ± 6.13	25.23 ± 6.38	22.37 ± 5.12	−0.310	[−0.918, 0.298]	0.318
Senior high school	24.13 ± 5.95	21.89 ± 4.95	26.53 ± 6.31	23.85 ± 6.00	0.429	[−0.121, 0.978]	0.126

SD, standard deviation. ^a^β is the coefficient of interaction term.

In terms of injury-related knowledge scores, primary school children improved by 4.147 points (95% CI: [3.821, 4.473], *p* < 0.001), junior school children improved by 1.837 points (95% CI: [1.565, 2.109], *p* < 0.001), and senior high school children improved by 5.212 points (95% CI: [4.950, 5.473], *p* < 0.001). Regarding injury-related belief scores, primary school children increased by 0.351 points (95% CI: [0.1890, 0.513], *p* < 0.001), senior high school children decreased by 0.169 points (95% CI: [−0.318, −0.020], *p* = 0.026), while the change in junior school children’s scores was not statistically significant (*β* = −0.023, 95% CI:[−0.184, 0.138], *p* = 0.783).

Regarding children’s injury-related protective and risky behaviors, DID analysis by grade level on changes in injury-related protective behavior scores before and after intervention revealed significant increases among primary school children (*β* = 7.139, 95% CI: [6.633, 7.645], *p* < 0.001), junior school children (*β* = 2.906, 95% CI: [2.466, 3.346], *p* < 0.001), and senior high school children (*β* = 4.540, 95% CI: [4.099, 4.980], *p* < 0.001). For injury-related risky behaviors, DID analysis showed a significant reduction in primary school children in the intervention group compared with the control group (*β* = −0.801, 95% CI: [−1.374, −0.228], *p* = 0.006), while no significant differences were found in junior school children (*β* = −0.310, 95% CI: [−0.918, 0.298], *p* = 0.318) or senior high school children (*β* = 0.429, 95% CI: [−0.121, 0.978], *p* = 0.126).

### 3.4 Parents’ knowledge, beliefs, and behaviors

Regarding parents’ knowledge and beliefs about injury prevention, the intervention group had baseline mean scores of 9.75 and 8.34, which increased to 14.05 and 8.95 at follow-up, respectively; both changes were statistically significant (all *p* < 0.001). In the control group, baseline scores were 9.78 and 8.51, with follow-up scores of 11.01 and 9.18, respectively, and these improvements also reached statistical significance for both knowledge and beliefs (all *p* < 0.001). DID analysis showed a significant difference in parental injury prevention knowledge between the intervention and control groups (*β* = 3.076, 95% CI: [2.804, 3.347], *p* < 0.001), whereas no significant difference was observed in beliefs (*β* = −0.061, 95% CI: [−0.276, 0.153], *p* = 0.576).

**Figure 3:**
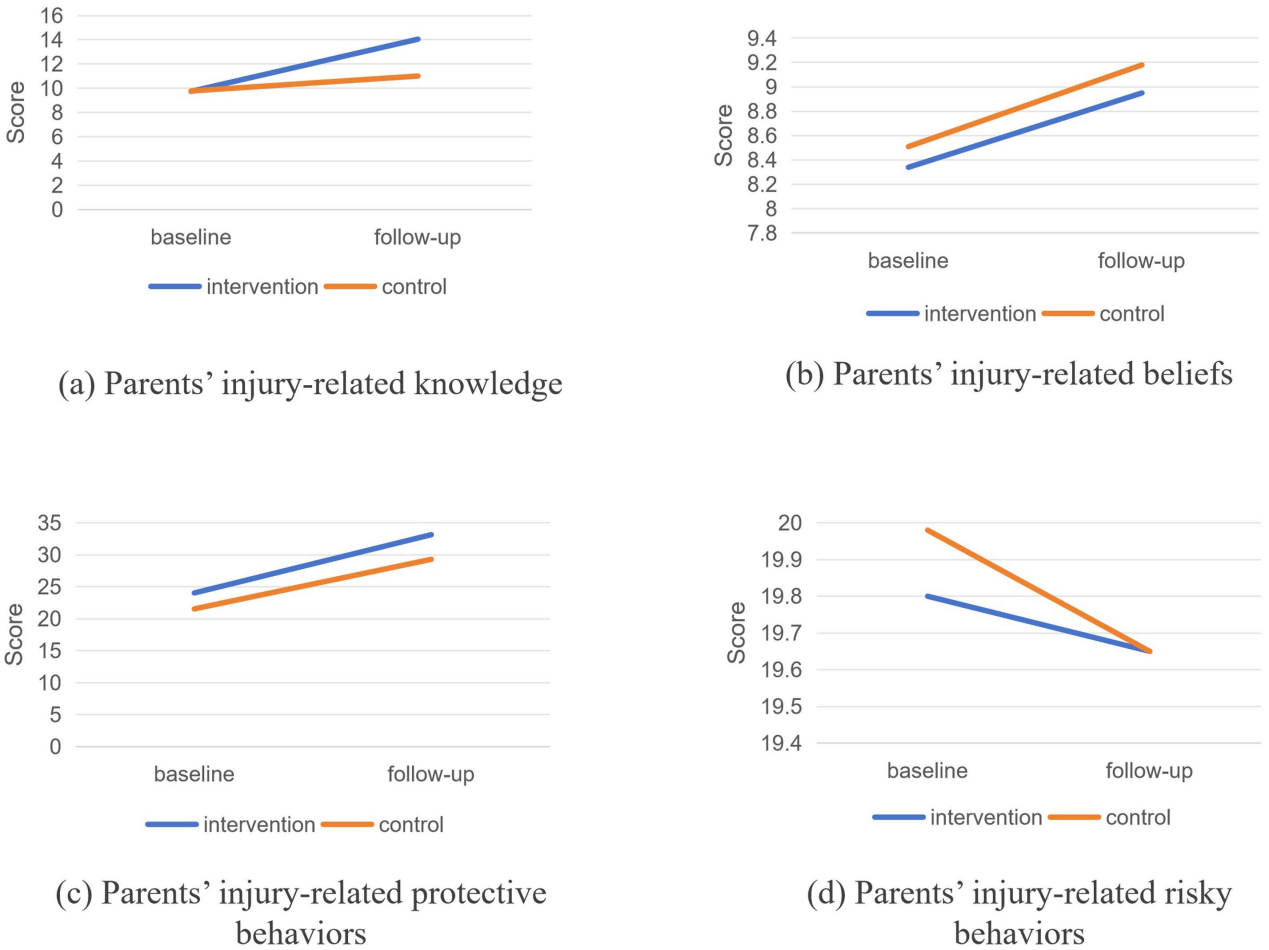
Comparison of baseline and follow-up between parents in the intervention and control groups.

For parents’ injury-related protective and risky behaviors, the intervention group achieved baseline scores of 24.03 and 19.80, with follow-up scores of 33.14 and 19.65, respectively; all changes were statistically significant (all *p* < 0.001). In the control group, baseline scores were 21.54 and 19.98, and follow-up scores were 29.29 and 19.65, respectively, with significant differences found for both behaviors (all *p* < 0.001). According to the DID analysis results, a significant difference was noted in parental protective behaviors (*β* = 1.358, 95% CI: [0.543, 2.174], *p* = 0.001), but no significant difference existed in risky behaviors (*β* = 0.180, 95% CI: [−0.263, 0.622], *p* = 0.426) (Table 4; Figure 3).

**Table 4:** The effectiveness of parents’ secondary outcome.

Variable	Intervention group		Control group		*β^a^*	95%CI	*p* value
	Baseline	Follow-up	Baseline	Follow-up			
Injury-related knowledge, mean ± SD	9.75 ± 2.92	14.05 ± 3.89	9.45 ± 2.49	11.01 ± 2.51	3.076	[2.804, 3.347]	<0.001
Injury-related beliefs, mean ± SD	8.34 ± 2.54	8.95 ± 2.43	8.51 ± 2.17	9.18 ± 2.06	−0.061	[−0.276, 0.153]	0.576
Injury-related protective behaviors, mean ± SD	28.26 ± 8.60	36.20 ± 10.68	21.54 ± 8.32	29.29 ± 9.68	1.358	[0.543, 2.174]	0.001
Injury-related risky behaviors, mean ± SD	19.80 ± 5.01	19.65 ± 4.10	19.98 ± 4.94	19.65 ± 4.24	0.180	[−0.263, 0.622]	0.426

SD standard deviation. ^a^β is the coefficient of interaction term.

## 4 Discussion

This study employed a cluster randomized controlled trial design to demonstrate the effectiveness of integrated interventions—combining enhanced child supervision, health education, and environmental improvement—in reducing childhood injury rates while improving children’s knowledge of injury prevention and their protective behaviors. However, no statistically significant difference was observed between the intervention and control groups regarding injury-related attitudes. This finding underscored the resistance of established attitudes to short-term interventions, highlighting the need for prolonged or targeted strategies to modify injury-related belief systems (10, 20).

The results indicated that the incidence of injuries in the intervention group decreased by 0.13%, while that in the control group increased by 2.00%. The rise in injury incidence among the control group may be attributed to the high randomness of childhood injury events, weakened awareness of injury risks due to the absence of intervention measures, and the prolonged follow-up period that exposed participants to greater external injury risk factors, leading to a natural increase in childhood injury incidence over time. In contrast, the implementation of intervention measures in the intervention group effectively suppressed the natural upward trend of childhood injury incidence and achieved a slight decrease, exerting a positive effect on preventing injury occurrence. From the perspective of health burden, childhood injuries rank among the leading causes of disability and death in children. Even a modest reduction in injury rates can significantly decrease emergency department visits, hospital stays, and long-term health needs, thereby alleviating pressure on pediatric healthcare resources. From the economic burden perspective, a decrease in childhood injury rates can reduce medical expenses, emergency care, and rehabilitation costs associated with injuries, alleviating the financial burden on families. It also minimizes lost workdays for parents caring for injured children and reduces future labor supply gaps caused by childhood disabilities.

Baseline data revealed that the injury-related knowledge scores of children and their parents in both the intervention and control groups were below 60%, whereas their belief scores exceeded 60%. This finding indicates that despite insufficient injury prevention knowledge, participants held accurate beliefs and positive attitudes toward injury prevention. Following the intervention, significant improvements in knowledge scores were observed among both parents and children. High school students exhibited the greatest increase, followed by parents of students in grades 1–3. In contrast, middle school children showed the least significant improvement in knowledge scores. The alignment between children’s cognitive development and intervention content may influence knowledge absorption efficiency. A study conducted in Jiangsu Province reported that the pass rate of injury awareness was 90.92% among high school students, 84.45% among primary school students, and 89.42% among parents—all higher than the 81.75% rate among junior high school students (21)—which is consistent with the findings of the present study. No substantial changes in belief scores were noted for either parents or children, with only a slight increase observed among upper primary school children (grades 4–6). Both children and parents demonstrated increased injury prevention protective behaviors, while a reduction in risky behaviors was only observed among upper primary school children (grades 4–6). This result is consistent with that of Mehreen et al. (22), who reported that younger children showed better performance in beliefs and injury-related risky behaviors compared to older peers.

This study focuses on children and adolescents aged 6–17 years. Compared with preschool children, this age group engages in activities across a wider range of settings, including family, school, and community environments. Thus, the development and implementation of a multi-dimensional child injury intervention model centered on the family-school-community triad are imperative. In China, basic community public services have been fully rolled out (23), laying a solid foundation for establishing cross-sectoral collaboration mechanisms for child injury prevention. Previous research has highlighted the critical role of parents and guardians in ensuring children’s safety (24), as their injury prevention-related knowledge, attitudes, and behaviors exert a significant influence on childhood injury risk. Therefore, for younger children, interventions were prioritized to strengthen parental engagement and capacity-building. Additionally, studies have confirmed the effectiveness of the Child-To-Child Approach in reducing childhood injuries and associated medical costs (25). Accordingly, our intervention strategy also adopted a child-centered approach, actively involving children in injury risk monitoring, health education initiatives, and environmental safety modifications. Such participation directly enhances children’s injury prevention KAP.

The combined interventions in this study resulted in a significant improvement in the knowledge of children and parents. The analysis of the improved knowledge scores of children of different ages suggests that, in future injury interventions, intervention toolkit should be designed to take into account the receptive and learning abilities of children of different ages, and more targeted interventions should be carried out. The present study found that injury-related beliefs and behaviors were resistant to change in the short term and were not significant in older children. These findings suggest that child injury prevention should be implemented on a long-term basis and integrated into the daily management of children’s health (26). Meanwhile, the intervention focused on modifying beliefs should be initiated among younger children to ensure optimal efficacy.

This study has several limitations. First, a questionnaire was used to collect data on childhood injuries occurring over the past 12 months, which may have introduced recall bias and thus affected the findings to a certain extent. Additionally, the injury data were not disaggregated by injury type, which may have influenced the study findings to some extent. Second, although significant improvements were observed in children’s injury prevention knowledge and protective behaviors, there was no corresponding significant reduction in childhood injury incidence. This indicates that a one-year intervention duration is insufficient to sustain long-term injury prevention effects, highlighting the need for extended intervention strategies. Third, this study did not explore the replicability of the intervention in other regions, nor did it examine the potential impacts of family background and socioeconomic factors on intervention outcomes.

## 5 Conclusion

This integrated intervention program, implemented in Shijiazhuang City for children aged 6 to 17, encompassing comprehensive childcare, health education and environmental improvements, has yielded positive outcomes in reducing child injury rates while enhancing both children’s and parents’ knowledge and behavioral capacity regarding injury prevention. This integrated family-school-community approach to injury prevention merits wider adoption. It overcomes the limitations of single-setting interventions, eliminates gaps in coverage, and facilitates resource complementarity, thereby enhancing the professionalism and sustainability of such programs.

## Data Availability

The raw data supporting the conclusions of this article will be made available by the authors, without undue reservation.
