# Supplementary material for: Effectiveness of enhanced supervision, health education and environmental improvement interventions for injuries among children aged 6–17 in Shijiazhuang
Source: Front Public Health. 2026 Feb 20;14:1733074. doi: 10.3389/fpubh.2026.1733074 (PMC12962914; doi:10.3389/fpubh.2026.1733074)
Supplement: Supplementary file 1 [file Table_1.docx]

**Table S1.**Child safety home checklist

| Area | Inspection items | True | False | Not applicable |
| --- | --- | --- | --- | --- |
| Sitting room | Furniture such as tables, coffee tables, etc. are protected at sharp corners. | □ | □ | □ |
|  | Heating equipment such as stoves, heaters, electric heaters, etc. are guarded. | □ | □ | □ |
|  | First aid number posted next to the telephone. | □ | □ | □ |
|  | Protective covers are placed on electrical outlets that children can touch. | □ | □ | □ |
|  | Kettles, thermos flasks, or drinking fountains should be out of children's reach. | □ | □ | □ |
|  | Window sills where children can climb should be guarded. | □ | □ | □ |
|  | Cabinets and drawers should have child locks. | □ | □ | □ |
| Bedroom | Balcony railings are high enough that children can't climb them. | □ | □ | □ |
|  | The windows are guarded so that children cannot climb on them. | □ | □ | □ |
|  | There are no tables, benches, etc. near the windows that children can climb on. | □ | □ | □ |
|  | Tables, cabinets, etc. are protected from sharp corners. | □ | □ | □ |
|  | Infants should sleep in a separate basket when sleeping in the same bed as their parents. | □ | □ | □ |
|  | Electrical sockets are protected from children's touch. | □ | □ | □ |
|  | Protect heating equipment such as stoves, heaters, electric heaters, etc. | □ | □ | □ |
|  | Ventilate the room, and install a blower in rooms with heating stoves. | □ | □ | □ |
| Children's room | The child's bed has guard rails with pins installed out of the child's reach. | □ | □ | □ |
|  | The floor around the child's bed is covered with soft protective material such as carpeting. | □ | □ | □ |
|  | There are no tables, benches or other furniture near the window that children can climb on. | □ | □ | □ |
|  | Windows are barred so that children cannot climb on them. | □ | □ | □ |
|  | Heating equipment such as stoves, heaters, electric heaters, etc. are guarded. | □ | □ | □ |
|  | Sundries should be kept in cupboards with doors and child locks. | □ | □ | □ |
| Kitchen | Set up a guardrail at the kitchen door, otherwise children will enter freely. | □ | □ | □ |
|  | Place pots and pans with the handles facing inward on the stovetop. | □ | □ | □ |
|  | Cover pots and pans when not cooking. | □ | □ | □ |
|  | Unplug microwave ovens and other electrical appliances when not in use. | □ | □ | □ |
|  | Extinguish the flame when the stove is not in use. | □ | □ | □ |
|  | Cover water tanks and buckets. | □ | □ | □ |
|  | Keep cupboards closed and lock them with child locks. | □ | □ | □ |
|  | Stack clutter neatly. | □ | □ | □ |
|  | Keep kettles, thermos flasks and drinking fountains out of children's reach. | □ | □ | □ |
|  | Gas should be protected by a burner switch. | □ | □ | □ |
|  | Keep knives and other sharp objects in cupboards with doors. | □ | □ | □ |
|  | Refrigerators should be closed and child-proofed. | □ | □ | □ |
| Restaurant | No tablecloths on dining tables and coffee tables. | □ | □ | □ |
|  | Protect dining room tables and coffee tables from sharp corners. | □ | □ | □ |
|  | Keep snacks such as peanuts, melon seeds and other nuts out of children's reach. | □ | □ | □ |
|  | Keep kettles and rice cookers out of children's reach. | □ | □ | □ |
|  | Protective covers for electrical sockets that children can touch. | □ | □ | □ |
|  | Child locks on cabinets. | □ | □ | □ |
| Bathroom | Grab bars in bathtubs or showers. | □ | □ | □ |
|  | Non-slip mats in bathtubs and showers. | □ | □ | □ |
|  | Unplug electrical appliances such as hairdryers when not in use. | □ | □ | □ |
|  | Don't store water in bathtubs, buckets, water tanks, etc. If water is stored, it should be covered. | □ | □ | □ |
|  | Put down the toilet lid when it is not in use. | □ | □ | □ |
|  | Keep razors out of children's reach and wrap blades when disposing of them. | □ | □ | □ |
|  | Unplug the washing machine when not in use. | □ | □ | □ |
|  | Protect electrical outlets that children can reach with protective covers. | □ | □ | □ |
|  | Keep toilet bowl cleaner in its original container and out of children's reach. | □ | □ | □ |
|  | Adequate lighting inside and outside the restroom. | □ | □ | □ |
|  | Prevent children from falling in when using the toilet or toilet seat. | □ | □ | □ |
|  | Keep floors clean and dry to prevent slipping. | □ | □ | □ |
| Courtyard | Have a working light in front of your home. | □ | □ | □ |
|  | Keep dogs vaccinated and on a leash. | □ | □ | □ |
|  | Rakes, shovels, and other sharp-edged tools are kept out of the reach of children. | □ | □ | □ |
|  | Stairs have handrails on at least one side. | □ | □ | □ |
|  | Pig pens, chicken coops, ponds, etc. have safety fences. | □ | □ | □ |
|  | Well platforms are raised and covered. | □ | □ | □ |
