# Supplementary material for: Effectiveness of enhanced supervision, health education and environmental improvement interventions for injuries among children aged 6–17 in Shijiazhuang
Source: Front Public Health. 2026 Feb 20;14:1733074. doi: 10.3389/fpubh.2026.1733074 (PMC12962914; doi:10.3389/fpubh.2026.1733074)
Supplement: Supplementary file 2 [file Table_2.docx]

**Table S2.**Safe Campus Checklist

| Area | Inspection items | True | False | Not applicable |
| --- | --- | --- | --- | --- |
| Sports ground | The sports ground is level with good drainage facilities and free of debris, nails, glass, wood and other hazards. | □ | □ | □ |
|  | Well covers and other fixed equipment are not buried above or below the level of the playing field. | □ | □ | □ |
|  | Sports equipments are stored in time after use. | □ | □ | □ |
|  | Basketball hoops, goal posts and other hardware equipment are equipped with soft cushions to prevent collisions. | □ | □ | □ |
|  | Formulas for the proper use of sports equipment or facilities are in place. | □ | □ | □ |
|  | Resilient flooring near sports equipment or facilities. | □ | □ | □ |
|  | All welding points of sports equipment or facilities are firm, screws are fixed without slack, and brackets and bolts are firm. | □ | □ | □ |
|  | The quality of the sports facilities is qualified and there is no deformation, corrosion, loosening, cracking, breaking and other quality problems. | □ | □ | □ |
|  | There is a certain safety distance between each sports facility. | □ | □ | □ |
| Safe electricity | There are no overhead wires or exposed wires on campus. | □ | □ | □ |
|  | The location and construction of power distribution devices are reasonable, safe and reliable, with measures to prevent accidental contact. | □ | □ | □ |
|  | Indoor wiring is concealed. | □ | □ | □ |
|  | Laboratory power sockets are located on laboratory tables. | □ | □ | □ |
|  | Power sockets use safety sockets with good safety performance. | □ | □ | □ |
|  | Installation of a power cut-off device at the power introduction point of each building. | □ | □ | □ |
| Safety precautions | Emergency evacuation facilities such as effective emergency evacuation routes, signs, emergency lights, etc. are available on campus. | □ | □ | □ |
|  | Fire extinguishers and fire alarms are available in laboratories, computer rooms and student dormitories. | □ | □ | □ |
|  | Fire hydrants in the building are of high quality and quantity. | □ | □ | □ |
|  | Fire extinguishers and fire fighting equipment are equipped as required. | □ | □ | □ |
|  | Burglar-proof doors and windows in key areas. | □ | □ | □ |
|  | Significant parts of the building are equipped with escape warnings. | □ | □ | □ |
|  | Doors and windows leading to the roof should be insured. | □ | □ | □ |
|  | Chemicals should be managed in accordance with security requirements. | □ | □ | □ |
|  | Fire fighting facilities and equipments should be added to the library. | □ | □ | □ |
|  | Fixed parking spaces for school motor vehicles and anti-theft measures should be taken. | □ | □ | □ |
| Management measure | Regular safety education for teachers and students. | □ | □ | □ |
|  | Have various safety management systems. | □ | □ | □ |
|  | Sign a safety responsibility letter at each level. | □ | □ | □ |
|  | Infirmary and medical staff meet the requirements. | □ | □ | □ |
|  | Emergency evacuation and other plans and regular drills. | □ | □ | □ |
|  | Injury prevention and control facilities and methods. | □ | □ | □ |
|  | There is a mechanism for regular inspection and maintenance of facilities. | □ | □ | □ |
