# Supplementary material for: Effectiveness of enhanced supervision, health education and environmental improvement interventions for injuries among children aged 6–17 in Shijiazhuang
Source: Front Public Health. 2026 Feb 20;14:1733074. doi: 10.3389/fpubh.2026.1733074 (PMC12962914; doi:10.3389/fpubh.2026.1733074)
Supplement: Supplementary file 3 [file Table_3.docx]

**Table S3.** The details of children’s questionnaire

| Question | Answer | Score |
| --- | --- | --- |
| Socio-demographic characteristics | | |
| 1.What is your gender? | ①male, ②female | — |
| 2.How old are you now? | years | — |
| 3.What is your grade level? | ①grade 1-3, ②grade 4-6, ③junior grade, ④senior grade | — |
| 4.During the past year, have you received treatment times diagnosed injury or missed more than one day of work, school, or rest due to injury? | ①True, ②False, ③Unknown | — |
| 5.How many unintentional injuries did you have in the past year? | ①1, ②2, ③3, ④4, ⑤>4 | — |
| Knowledge | | |
| 1.Injury is the first cause of death among teenagers. | ①True, ②False, ③Unknown | Correct answer=1,  Incorrect answer=0,  Unknown=0. |
| 2.When riding a private car, you need to wear a seat belt in the co-pilot position, but not in the back row. | ①True, ②False, ③Unknown |  |
| 3.When walking, you can use your mobile phone (answer phone calls, send text messages) or headphones (listen to music, etc.) | ①True, ②False, ③Unknown |  |
| 4.You should warm up before swimming. | ①True, ②False, ③Unknown |  |
| 5.Falling on the flat ground or falling from a short position will not cause serious injury. | ①True, ②False, ③Unknown |  |
| 6.Burn wounds caused by hydrothermal solution, steam and flame should be immediately washed or soaked in clean cold water for a period of time. | ①True, ②False, ③Unknown |  |
| 7.Applying toothpaste and soy sauce immediately after scalding can alleviate the injury. | ①True, ②False, ③Unknown |  |
| 8.Keep indoor ventilation when using gas water heater to take a bath. | ①True, ②False, ③Unknown |  |
| 9.If someone is found to have carbon monoxide poisoning, he should first open the window for ventilation or move it to an environment with fresh air circulation. | ①True, ②False, ③Unknown |  |
| 10.Don't disturb cats and dogs when they are eating or sleeping. | ①True, ②False, ③Unknown |  |
| 11.Wash the wound with running water immediately after being bitten by cats and dogs, and get rabies vaccine in time. | ①True, ②False, ③Unknown |  |
| 12.When someone drowns, if he swims well, he can immediately enter the water to save people. | ①True, ②False, ③Unknown |  |
| 13.When someone drowns, you can hold hands to save people. | ①True, ②False, ③Unknown |  |
| 14.What height do you think is enough to use an adult seatbelt? | ①1 meter, ②1.25 meters, ③1.45 meters, ④1.6 meters, ⑤Unknown |  |
| 15.How old do you think it is to ride a bike on the road? | ①≥4, ②≥6, ③≥10, ④≥12, ⑤Unknown |  |
| 16.The telephone number of the Women's Rights Protection Public Service Hotline set up by the All-China Women's Federation is: | telephone number:________ |  |
| 17.The telephone number of the Youth Psychological Counseling and Legal Aid Hotline set up by the Central Committee of the Communist Youth League is: | telephone number:________ |  |
| 18.Government public service hotlines that can provide disease prevention and control and health care are: | ①12315, ②12369, ③12320, ④12306, ⑤Unknown |  |
| 19.After finding someone injured, you should first when helping him to rescue: | ①Call for help, ②Stop the bleeding, ③Judge the injured person's breathing and heart rate, ④Judge the consciousness of the injured person, ⑤Ensure the safety of the environment and yourself, ⑥Unknown |  |
| 20.Typically, the ratio of chest compressions to artificial respiration when a person performs CPR is: | ①30:1, ②30:2, ③30:3, ④20:1, ⑤20:2, ⑥20:3, ⑦Unknown |  |
| Beliefs | | |
| 1.Injury is an accident and cannot be prevented. | ①Agree, ②Disagree, ③Unknown | Correct answer=1,  Incorrect answer=0,  Unknown=0. |
| 2.You should wear a helmet when riding or riding a motorcycle. | ①Agree, ②Disagree, ③Unknown |  |
| 3.If you are a good swimmer, you can go swimming alone. | ①Agree, ②Disagree, ③Unknown |  |
| 4.If accompanied by an adult, you can go swimming, playing or bathing in waters or places without lifeguards/lifesaving equipment. | ①Agree, ②Disagree, ③Unknown |  |
| 5.There will be bumps in play and sports, and you can't stand big injuries. | ①Agree, ②Disagree, ③Unknown |  |
| 6.If you have learned roller skating, there is no need to wear protective tools such as helmets and knee pads. | ①Agree, ②Disagree, ③Unknown |  |
| 7.You can set off fireworks with your hands. | ①Agree, ②Disagree, ③Unknown |  |
| 8.If the child is disobedient, parents can kick or hit him/her. | ①Agree, ②Disagree, ③Unknown |  |
| 9.After a student is injured, only professional technicians can give first aid. | ①Agree, ②Disagree, ③Unknown |  |
| Behaviors | | |
| 1.When playing and exercising outdoors (in communities, sports venues, amusement places, etc.), the environment for playing and exercising will be checked for safety. | ①Never, ②Rarely, ③Sometimes, ④Often, ⑤Always, ⑥Not applicable | ①Never=1,  ②Rarely=2,  ③Sometimes=3,  ④Often=4,  ⑤Always=5,  ⑥Not applicable=0. |
| 2.Failing to tell parents and/or teachers after the injury. | ①Never, ②Rarely, ③Sometimes, ④Often, ⑤Always, ⑥Not applicable |  |
| 3.Pushing and slapping on the balcony/roof railing or on the stairs | ①Never, ②Rarely, ③Sometimes, ④Often, ⑤Always, ⑥Not applicable |  |
| 4.Climb roofs, windows, guardrails, trees, etc. | ①Never, ②Rarely, ③Sometimes, ④Often, ⑤Always, ⑥Not applicable |  |
| 5.Wear protective gear for skateboarding or roller skating. | ①Never, ②Rarely, ③Sometimes, ④Often, ⑤Always, ⑥Not applicable |  |
| 6.Sit on or slide down the handrail. | ①Never, ②Rarely, ③Sometimes, ④Often, ⑤Always, ⑥Not applicable |  |
| 7.Play with matches, lighters and other ignition appliances. | ①Never, ②Rarely, ③Sometimes, ④Often, ⑤Always, ⑥Not applicable |  |
| 8.Contact with flammable materials, etc. | ①Never, ②Rarely, ③Sometimes, ④Often, ⑤Always, ⑥Not applicable |  |
| 9.Put cold water before hot water when taking a bath or washing feet. | ①Never, ②Rarely, ③Sometimes, ④Often, ⑤Always, ⑥Not applicable |  |
| 10.Taking drugs with incomplete or scattered packages. | ①Never, ②Rarely, ③Sometimes, ④Often, ⑤Always, ⑥Not applicable |  |
| 11.Pick wild fruits to eat | ①Never, ②Rarely, ③Sometimes, ④Often, ⑤Always, ⑥Not applicable |  |
| 12.Close contact with strange dogs or kittens. | ①Never, ②Rarely, ③Sometimes, ④Often, ⑤Always, ⑥Not applicable |  |
| 13.Fight with others (one or more people) | ①Never, ②Rarely, ③Sometimes, ④Often, ⑤Always, ⑥Not applicable |  |
| 14.Make fun of other people's physical defects or looks. | ①Never, ②Rarely, ③Sometimes, ④Often, ⑤Always, ⑥Not applicable |  |
| 15.Being teased for one's physical defects or looks. | ①Never, ②Rarely, ③Sometimes, ④Often, ⑤Always, ⑥Not applicable |  |
| 16.Be asked for property. | ①Never, ②Rarely, ③Sometimes, ④Often, ⑤Always, ⑥Not applicable |  |
| 17.Don't obey the traffic lights when crossing the road. | ①Never, ②Rarely, ③Sometimes, ④Often, ⑤Always, ⑥Not applicable |  |
| 18.Use a mobile phone (answer phone calls, send text messages) or headphones (listen to music, etc.) while walking. | ①Never, ②Rarely, ③Sometimes, ④Often, ⑤Always, ⑥Not applicable |  |
| 19.Ride on the motorway | ①Never, ②Rarely, ③Sometimes, ④Often, ⑤Always, ⑥Not applicable |  |
| 20.Before swimming or paddling, do necessary warm-up exercises. | ①Never, ②Rarely, ③Sometimes, ④Often, ⑤Always, ⑥Not applicable |  |
| 21.Swimming, playing or bathing in waters or places without lifeguards/life-saving equipment without an adult. | ①Never, ②Rarely, ③Sometimes, ④Often, ⑤Always, ⑥Not applicable |  |
