# Supplementary material for: Effectiveness of enhanced supervision, health education and environmental improvement interventions for injuries among children aged 6–17 in Shijiazhuang
Source: Front Public Health. 2026 Feb 20;14:1733074. doi: 10.3389/fpubh.2026.1733074 (PMC12962914; doi:10.3389/fpubh.2026.1733074)
Supplement: Supplementary file 4 [file Table_4.docx]

**Table S4.** The details of parents’ questionnaire

| Question | Answer | Score |
| --- | --- | --- |
| Socio-demographic characteristics | | |
| 1.What is your relationship with child? | ①Mother, ②Father, ③Other (Investigation terminated) | — |
| 2.Child sex | ①male, ②female | — |
| 3.Child age | years | — |
| 4.What is your child grade level? | ①grade 1-3, ②grade 4-6, ③junior grade, ④senior grade | — |
| 5.During the past year, have your child received treatment times diagnosed injury or missed more than one day of work, school, or rest due to injury? | ①True, ②False, ③Unknown | — |
| 6.How many unintentional injuries did your child have in the past year? | ①1, ②2, ③3, ④4, ⑤>4 | — |
| Knowledge | | |
| 1.Injury is the first cause of death among teenagers. | ①True, ②False, ③Unknown | Correct answer=1,  Incorrect answer=0,  Unknown=0. |
| 2.When riding a private car, you need to wear a seat belt in the co-pilot position, but not in the back row. | ①True, ②False, ③Unknown |  |
| 3.When riding, children should use different types of child safety seats according to their age and weight. | ①True, ②False, ③Unknown |  |
| 4.Children should warm up before swimming. | ①True, ②False, ③Unknown |  |
| 5.Furniture that children can easily climb cannot be placed near balconies and windows at home. | ①True, ②False, ③Unknown |  |
| 6.Falling on the flat ground or falling from a short position will not cause serious injury. | ①True, ②False, ③Unknown |  |
| 7.Horizontal guardrail is safer. | ①True, ②False, ③Unknown |  |
| 8.Burn wounds caused by hydrothermal solution, steam and flame should be immediately washed or soaked in clean cold water for a period of time | ①True, ②False, ③Unknown |  |
| 9.Applying toothpaste and soy sauce immediately after scalding can alleviate the injury. | ①True, ②False, ③Unknown |  |
| 10.Cleaning agents, chemicals, medicines, etc. should be placed out of the reach of children. | ①True, ②False, ③Unknown |  |
| 11.Keep indoor ventilation when using gas water heater to take a bath. | ①True, ②False, ③Unknown |  |
| 12.If someone is found to have carbon monoxide poisoning, he should first open the window for ventilation or move it to an environment with fresh air circulation. | ①True, ②False, ③Unknown |  |
| 13.When someone drowns, children can hold hands to save people. | ①True, ②False, ③Unknown |  |
| 14.What height do you think is enough to use an adult seatbelt? | ①1 meter, ②1.25 meters, ③1.45 meters, ④1.6 meters, ⑤Unknown |  |
| 15.How old do you think it is to ride a bike on the road? | ①≥4, ②≥6, ③≥10, ④≥12, ⑤Unknown |  |
| 16.The telephone number of the Youth Psychological Counseling and Legal Aid Hotline set up by the Central Committee of the Communist Youth League is: | telephone number:________ |  |
| 17.Government public service hotlines that can provide disease prevention and control and health care are: | ①12315, ②12369, ③12320, ④12306, ⑤Unknown |  |
| 18.After finding someone injured, you should first when helping him to rescue | ①Call for help, ②Stop the bleeding, ③Judge the injured person's breathing and heart rate, ④Judge the consciousness of the injured person, ⑤Ensure the safety of the environment and yourself, ⑥Unknown |  |
| 19.Typically, the ratio of chest compressions to artificial respiration when a person performs CPR is: | ①30:1, ②30:2, ③30:3, ④20:1, ⑤20:2, ⑥20:3, ⑦Unknown |  |
| Beliefs | | |
| 1.Injury is an accident and cannot be prevented. | ①Agree, ②Disagree, ③Unknown | Correct answer=1,  Incorrect answer=0,  Unknown=0. |
| 2.You can do something else while looking after the children. | ①Agree, ②Disagree, ③Unknown |  |
| 3.Adults can let older children look after children when they are busy. | ①Agree, ②Disagree, ③Unknown |  |
| 4.Children should wear helmets when riding or riding motorcycles. | ①Agree, ②Disagree, ③Unknown |  |
| 5.Children will stumble in play and sports, and they can't stand big injuries. | ①Agree, ②Disagree, ③Unknown |  |
| 6.The child's body grows fast, and the sports protective equipment bought now will not be suitable for a long time. Let's buy it later. | ①Agree, ②Disagree, ③Unknown |  |
| 7.If your child is a good swimmer, he/she can go swimming alone. | ①Agree, ②Disagree, ③Unknown |  |
| 8.If accompanied by an adult, children can go swimming, playing and bathing in places where lifeguards/lifesaving equipment are not available. | ①Agree, ②Disagree, ③Unknown |  |
| 9.Don't disturb cats and dogs when they are eating or sleeping. | ①Agree, ②Disagree, ③Unknown |  |
| 10.Wash the wound with running water immediately after being bitten by cats and dogs, and get rabies vaccine in time. | ①Agree, ②Disagree, ③Unknown |  |
| 11.If the child is disobedient, parents can kick or hit him/her. | ①Agree, ②Disagree, ③Unknown |  |
| 12.After the child is injured, only professional and technical personnel can give first aid. | ①Agree, ②Disagree, ③Unknown |  |
| Behaviors | | |
| 1.Leave the child alone for half an hour. | ①Never, ②Rarely, ③Sometimes, ④Often, ⑤Always, ⑥Not applicable | ①Never=1,  ②Rarely=2,  ③Sometimes=3,  ④Often=4,  ⑤Always=5,  ⑥Not applicable=0. |
| 2.Leave the child alone with other children for half an hour. | ①Never, ②Rarely, ③Sometimes, ④Often, ⑤Always, ⑥Not applicable |  |
| 3.Let the children stand in the shopping cart when shopping. | ①Never, ②Rarely, ③Sometimes, ④Often, ⑤Always, ⑥Not applicable |  |
| 4.When playing and exercising with children outdoors (in communities, sports venues, amusement places, etc.), the safety of children's playing and exercising environment will be checked. | ①Never, ②Rarely, ③Sometimes, ④Often, ⑤Always, ⑥Not applicable |  |
| 5.Ask children to warm up before exercise. | ①Never, ②Rarely, ③Sometimes, ④Often, ⑤Always, ⑥Not applicable |  |
| 6.Wear protective tools (such as knee pads, elbow pads, wrist pads, helmets, etc.) for children during roller skating and other activities. | ①Never, ②Rarely, ③Sometimes, ④Often, ⑤Always, ⑥Not applicable |  |
| 7.Keep the children in the kitchen. | ①Never, ②Rarely, ③Sometimes, ④Often, ⑤Always, ⑥Not applicable |  |
| 8.Put matches, lighters and other ignition appliances or hot water containers, hot food, etc. where children can get them. | ①Never, ②Rarely, ③Sometimes, ④Often, ⑤Always, ⑥Not applicable |  |
| 9.Put cold water before hot water when taking a bath or washing feet. | ①Never, ②Rarely, ③Sometimes, ④Often, ⑤Always, ⑥Not applicable |  |
| 10.Put detergent, pesticides and other daily chemicals in empty beverage bottles. | ①Never, ②Rarely, ③Sometimes, ④Often, ⑤Always, ⑥Not applicable |  |
| 11.Keep drugs/toxic substances indoors where children can get them. | ①Never, ②Rarely, ③Sometimes, ④Often, ⑤Always, ⑥Not applicable |  |
| 12.Allow children to have close contact with strange small animals. | ①Never, ②Rarely, ③Sometimes, ④Often, ⑤Always, ⑥Not applicable |  |
| 13.Don't obey the traffic lights when crossing the road with your children. | ①Never, ②Rarely, ③Sometimes, ④Often, ⑤Always, ⑥Not applicable |  |
| 14.When walking with children, let children wear bright colors or reflective clothes, schoolbags and other accessories. | ①Never, ②Rarely, ③Sometimes, ④Often, ⑤Always, ⑥Not applicable |  |
| 15.When walking with children, use mobile phones (answering phone calls, sending text messages) or headphones (listening to music, etc.) while walking. | ①Never, ②Rarely, ③Sometimes, ④Often, ⑤Always, ⑥Not applicable |  |
| 16.When riding a bicycle with children, ride on the motorway or sidewalk. | ①Never, ②Rarely, ③Sometimes, ④Often, ⑤Always, ⑥Not applicable |  |
| 17.When riding a bicycle with children, wear a helmet for the children. | ①Never, ②Rarely, ③Sometimes, ④Often, ⑤Always, ⑥Not applicable |  |
| 18.When taking children with you in a private car, let them use seat belts/safety seats. | ①Never, ②Rarely, ③Sometimes, ④Often, ⑤Always, ⑥Not applicable |  |
| 19.Dump/discharge the water in containers that can store water, such as pots, barrels, washing machines and toilets, in time after using them. | ①Never, ②Rarely, ③Sometimes, ④Often, ⑤Always, ⑥Not applicable |  |
| 20.Cover water storage containers such as water storage buckets and water tanks at home with covers that children can't easily open. | ①Never, ②Rarely, ③Sometimes, ④Often, ⑤Always, ⑥Not applicable |  |
| 21.Take your children swimming, playing and bathing in waters or places where lifeguards/lifesaving equipment are not available. | ①Never, ②Rarely, ③Sometimes, ④Often, ⑤Always, ⑥Not applicable |  |
