# Supplementary material for: Effectiveness of enhanced supervision, health education and environmental improvement interventions for injuries among children aged 6–17 in Shijiazhuang
Source: Front Public Health. 2026 Feb 20;14:1733074. doi: 10.3389/fpubh.2026.1733074 (PMC12962914; doi:10.3389/fpubh.2026.1733074)
Supplement: Supplementary file 5 [file Table_5.docx]

**Table S5.** The effectiveness of secondary outcome within each group

| **Variable** | **Intervention group** | | |  | **Control group** | | |
| --- | --- | --- | --- | --- | --- | --- | --- |
|  | **Baseline** | **Follow-up** | ***P* value** |  | **Baseline** | **Follow-up** | ***P* value** |
| Injury-related knowledge, mean±SD |  |  |  |  |  |  |  |
| Parents | 9.75±2.92 | 14.05±3.89 | < 0.001 |  | 9.78±2.49 | 11.01±2.51 | < 0.001 |
| Children | 9.84±2.86 | 12.86±3.86 | < 0.001 |  | 10.02±2.44 | 10.21±2.38 | < 0.001 |
| Primary school | 9.44±2.79 | 12.96±3.65 | < 0.001 |  | 10.57±2.91 | 9.94±2.54 | < 0.001 |
| Junior school | 9.12±2.83 | 10.87±3.09 | < 0.001 |  | 9.77±2.38 | 9.69±2.30 | 0.327 |
| Senior high school | 9.02±2.67 | 14.88±3.68 | < 0.001 |  | 9.99±2.06 | 10.64±2.24 | < 0.001 |
| Injury-related beliefs, mean±SD |  |  |  |  |  |  |  |
| Parents | 8.34±2.54 | 8.95±2.43 | < 0.001 |  | 8.51±2.17 | 9.18±2.06 | < 0.001 |
| Children | 6.80±1.82 | 6.97±1.44 | < 0.001 |  | 7.13±1.54 | 7.34±1.50 | < 0.001 |
| Primary school | 6.80±1.71 | 7.12±1.34 | < 0.001 |  | 7.06±1.47 | 7.02±1.49 | 0.617 |
| Junior school | 6.67±1.72 | 6.80±1.58 | 0.037 |  | 7.04±1.60 | 7.19±1.48 | 0.006 |
| Senior high school | 6.88±1.98 | 7.02±1.35 | 0.026 |  | 7.30±1.50 | 7.60±1.47 | < 0.001 |
| Injury-related protective behaviors, mean±SD |  |  |  |  |  |  |  |
| Parents | 24.03±8.53 | 33.14±9.20 | < 0.001 |  | 21.54±8.32 | 29.29±9.68 | < 0.001 |
| Children | 13.15±3.75 | 17.53±5.78 | < 0.001 |  | 14.13±4.08 | 13.93±4.40 | 0.026 |
| Primary school | 13.17±4.11 | 19.13±5.42 | < 0.001 |  | 15.84±4.40 | 14.67±4.65 | < 0.001 |
| Junior school | 13.68±3.44 | 16.02±5.29 | < 0.001 |  | 13.74±3.95 | 13.18±4.03 | < 0.001 |
| Senior high school | 12.75±3.54 | 17.76±6.16 | < 0.001 |  | 13.43±3.67 | 13.91±4.37 | < 0.001 |
| Injury-related risky behaviors, mean±SD |  |  |  |  |  |  |  |
| Parents | 19.80±5.01 | 19.65±4.10 | < 0.001 |  | 19.98±4.94 | 19.65±4.24 | < 0.001 |
| Children | 23.68±5.95 | 21.74±5.61 | < 0.001 |  | 24.59±6.45 | 22.64±5.65 | < 0.001 |
| Primary school | 22.24±5.60 | 21.50±5.67 | < 0.001 |  | 20.72±4.99 | 20.79±4.88 | 0.757 |
| Junior school | 24.96±6.00 | 21.79±6.13 | < 0.001 |  | 25.23±6.38 | 22.37±5.12 | < 0.001 |
| Senior high school | 24.13±5.95 | 21.89±4.95 | < 0.001 |  | 26.53±6.31 | 23.85±6.00 | < 0.001 |

Abbreviations: *P* value is the result of comparing baseline with follow-up data in intervention group and control group.
